# Revealing the developmental origin and lineage predilection of neural progenitors within human bone marrow via single-cell analysis: implications for regenerative medicine

**DOI:** 10.1186/s13073-023-01224-0

**Published:** 2023-09-04

**Authors:** Zhang Changmeng, Wang Hongfei, Martin Chi-Hang Cheung, Ying-Shing Chan, Graham Ka-Hon Shea

**Affiliations:** 1 Department of Orthopaedics and Traumatology, School of Clinical Medicine, Li Ka Shing Faculty of Medicine, The University of Hong Kong, Pokfulam, Hong Kong; 2 School of Biomedical Sciences, Li Ka Shing Faculty of Medicine, The University of Hong Kong, Pokfulam, Hong Kong

**Keywords:** Neural crest, Bone marrow stromal cell, Cell differentiation, Single-cell RNA sequencing, Regenerative medicine, Cell lineage

## Abstract

**Background:**

Human bone marrow stromal cells (BMSCs) are an easily accessible and expandable progenitor population with the capacity to generate neural cell types in addition to mesoderm. Lineage tracing studies in transgenic animals have indicated Nestin + BMSCs to be descended from the truncal neural crest. Single-cell analysis provides a means to identify the developmental origin and identity of human BMSC-derived neural progenitors when lineage tracing remains infeasible. This is a prerequisite towards translational application.

**Methods:**

We attained transcriptomic profiles of embryonic long bone, adult human bone marrow, cultured BMSCs and BMSC-derived neurospheres. Integrated scRNAseq analysis was supplemented by characterization of cells during culture expansion and following provision of growth factors and signalling agonists to bias lineage.

**Results:**

Reconstructed pseudotime upon the integrated dataset indicated distinct neural and osteogenic differentiation trajectories. The starting state towards the neural differentiation trajectory consisted of Nestin + /MKI67 + BMSCs, which could also be diverted towards the osteogenic trajectory via a branch point. Nestin + /PDGFRA + BMSCs responded to neurosphere culture conditions to generate a subpopulation of cells with a neuronal phenotype according to marker expression and gene ontogeny analysis that occupied the end state along the neural differentiation trajectory. Reconstructed pseudotime also revealed an upregulation of BMP4 expression during culture of BMSC-neurospheres. This provided the rationale for culture supplementation with the BMP signalling agonist SB4, which directed progenitors to upregulate Pax6 and downregulate Nestin.

**Conclusions:**

This study suggested BMSCs originating from truncal neural crest to be the source of cells within long bone marrow possessing neural differentiation potential. Unravelling the transcriptomic dynamics of BMSC-derived neural progenitors promises to enhance differentiation efficiency and safety towards clinical application in cell therapy and disease modelling.

**Supplementary Information:**

The online version contains supplementary material available at 10.1186/s13073-023-01224-0.

## Background

Traumatic and degenerative diseases affecting the nervous system result in functional impairment following the loss of neurons and glia. Cell replacement therapy may be of benefit, and within the context of traumatic spinal cord injury, there is abundant preclinical evidence indicating that transplantation of neural progenitors accelerates regeneration [1]. Likewise in animal models of multiple sclerosis, neural progenitor transplantation replenishes the oligodendrocyte population and facilitates remyelination [2]. One of the obstacles to translation is the lack of a readily accessible and expandible neural progenitor source. Neural stem cells reside within the subventricular zone of the brain or ependymal region of the spinal cord and therefore are not easily harvested. Neural stem and progenitor cells generated from embryonic stem (ES) cells and inducible pluripotent stem cells (iPSCs) remain hindered by ethical constraints [3] and genetic instability [4] respectively.

Bone marrow stromal cells (BMSCs) are somatic stem cells initially characterized to give rise to mesodermal tissues such as bone, cartilage and fat [5]. Ease of isolation and culture as well as demonstration of differentiation potential beyond the mesodermal lineage are aspects favouring their clinical application for autologous cell therapy and disease modelling [6]. BMSCs exhibit the potential for neural differentiation towards glia and neurons when growth factors are provided for sequentially under defined culture protocols [7, 8]. As evidence for genetic instability of iPSCs has become apparent, derivation of autologous cell types in the absence of genetic manipulation provides a unique translational advantage [4].

An explanation for the neural differentiation potential of BMSCs is that a cellular subpopulation is derived from the neural crest [9, 10]. Lineage tracing studies in the rodent demonstrate that neural crest stem cells (NCSCs) migrate to long bone marrow by way of developing vasculature. These neural crest progenitors may be expanded as neurospheres and generate both neural and mesenchymal cell types [9]. Nestin + /PDGFRA − neural crest-derived BMSCs within long bone have been shown to give rise to glia, whilst a distinct Nestin + /CXCL12 + subpopulation establishes the haematopoietic stem cell (HSC) niche via chemokine secretion [10]. It is assumed that neural progenitors obtained from human bone marrow have a similar identity and developmental origin, yet work characterizing NCSC-like progenitors within human bone marrow is lacking.

Formation of neural crest (NC) over the neural plate border results from complex interplay between signalling factors, stage-specific transcription factors and a synchrony of complex downstream regulatory network events [11]. Advances in single-cell RNA sequencing (scRNAseq) have allowed for comparative transcriptomic analysis of heterogenous progenitor populations towards understanding developmental relationships [12]. By segmenting the transcriptional information of individual cells, subpopulations of distinct developmental origin and differentiation potential may be identified. Towards characterization, neural crest-derived progenitors residing within human bone marrow would be expected to possess a post-migratory phenotype. Post-migratory NCSCs express Nestin [10], Snail1/2, Sox9 [13] and Twist1 [9] and PRRX1 [14–16].

Here, we performed comparative transcriptomic analysis upon scRNAseq datasets obtained from human embryonic long bone, adult long bone marrow, cultured human BMSCs and BMSC-derived neurospheres. Cell fate trajectories and gene regulatory networks were elucidated. Cultured cells represented intermediary progenitor states generated from neural induction protocol towards application in regenerative medicine [7–9]. Our findings provide an integrative understanding of neural progenitors within human bone marrow and promise to improve cell selection and differentiation efficiency towards safe clinical application.

## Methods

### Samples

This study was approved by the Institutional Review Board of the University of Hong Kong (Study No. UW 19–864). Reamed bone marrow was obtained from three consenting donors (41-year-old female, 28-year-old male, 33-year-old male) receiving intramedullary nailing for femoral fractures. Two public scRNAseq datasets were obtained from the Gene Expression Omnibus (GEO) which contained transcriptomic data of long bones from three human embryos at 5–8 weeks post-conception (GSE143753 [17]), and from femoral head bone marrow (GSE147287 [18]) of two healthy donors (84-year-old male, 67-year-old female).

### Human BMSC and neurosphere culture

Culture of BMSCs was performed in accordance with our previous publication [8]. Briefly, reamed human bone marrow was resuspended in growth medium containing αMEM supplemented with 15% foetal bovine serum (FBS) and seeded on tissue culture plates. Non-adherent cells were removed following medium exchange 48 h later. BMSCs were maintained in growth medium until reaching 80% confluency, then detached with TrypLE Express and passaged in a 1:2 ratio. For neurosphere formation, Passage 5 (P5) BMSCs were seeded at a density of 50,000 cells per well onto Ultra Low® non-adherent 6-well plates (Corning) in sphere-forming medium comprising of neurobasal medium supplemented with 2% B27, 1% GlutaMAX, 20 ng/ml bFGF2 and 20 ng/ml EGF. To bias differentiation, 1 μM SB4 was added to sphere-forming medium in the SB4 treatment group. Neurospheres were maintained for 8 days with culture medium exchanged every 3 days.

### Single-cell RNA sequencing

P5 BMSCs and day 8 BMSC-derived neurospheres (both generated from the 41-year-old female donor) were dissociated into single cells by treatment with papain at 37 °C for 30 min. Dissociated cells were filtered through a 40-μm cell strainer with determination of cell concentration and viability (> 98%) upon chamber slides following trypan blue staining. Cells were resuspended in ice-cold PBS and 0.04% bovine serum albumin at a density of 1000 cells/μl. Resuspended BMSCs and BMSC-derived neurospheres were loaded onto the 10 × Genomics Chromium platform according to the manufacturer’s protocol, whilst scRNAseq libraries were prepared using the Chromium Single Cell 3ʹ GEM Library and Gel Bead Kit v3. All libraries were sequenced on the Illumina Novaseq 6000 platform using a S4 flow cell and PE151 kit.

### scRNAseq data preprocessing

Illumina base call files (BCL) of cultured BMSCs and BMSC-derived neurospheres were demultiplexed and converted to FASTQ files with the bcl2fastq2 programme (version 2.20, Illumina). SRA format data of three human embryonic long bone samples (GSM4274191, GSM4274192, GSM4274193) [17] and two fresh human bone marrow samples (GSM4423510, GSM4423511) [18] were acquired and converted to FASTQ files with the SRA Toolkit (version 3.0.1, https://github.com/ncbi/sra-tools/wiki). The FASTQ files were then aligned to the human genome reference sequence (GRCh38) for filtering, barcode counting and UMI counting by the Cell Ranger (v7.1, 10X Genomics) count pipeline. A barcode table, gene table and gene expression matrix was generated for downstream analyses.

### Seurat for quality control and pre-processing

Seurat R package (v4.1.2) was utilized for downstream quality control and clustering [19]. Count matrix was read as a Seurat object, and cells with less than 200 genes expressed discarded, as were genes which were detected in less than 3 cells. A total of seven Seurat objects were created and comprised of three human embryonic long bone samples (GSM4274191, GSM4274192, GSM4274193) [17], two fresh human bone marrow samples (GSM4423510, GSM4423511) [18], one cultured BMSC sample and one BMSC-derived neurosphere sample. We filtered out low-quality cells with reference to the following criteria: feature counts > 2000 or < 5000 and mitochondrial counts < 5% for human embryonic long bones, feature counts > 200 or < 7000 and mitochondrial counts < 5% for fresh BMSCs, feature counts > 5000 or < 3000 and mitochondrial counts < 20% for cultured BMSCs, and feature counts > 3000 or < 8000 and mitochondrial counts < 20% for BMSC-derived neurospheres. We attained 18,324 cells from human embryonic long bones, 18,133 cells from fresh human bone marrow, 6091 cells from cultured BMSCs and 10,249 cells from BMSC-derived neurosphere cells for analysis. In addition, we isolated in silico a BMSC subpopulation amongst fresh human bone marrow (fresh BMSCs) consisting of 4052 cells by using LEPR^hi^/PDGFRA^hi^/ CD45^low^ as screening markers [20]. A Log Normalization method was employed via the log-transform operation and a scale factor of 10,000. Top 2000 highly variable genes (HVGs) were selected for downstream analysis.

### Data integration and analysis of individual scRNAseq datasets

Four datasets (human embryonic long bones, cultured BMSCs, fresh BMSCs and BMSC-neurospheres) were integrated using Seurat in an anchor-based workflow with CCA dimension reduction and using 2000 variable features for anchor identification. For the integrated data set, k-Nearest Neighbour (KNN) graphs were constructed using the top 20 PCs, and 14 clusters were identified using Louvain graph-clustering with a resolution of 0.8. For individual analyses, the top 10 PCs were selected to construct KNN graphs. We embedded the graph in a two-dimensional visualization space using UMAP with a resolution of 0.5 for fresh human bone marrow, and a resolution of 0.3 for embryonic long bone, cultured BMSCs, BMSC-derived neurospheres and fresh BMSCs. Subsequently, “FindAllMarkers” function in Seurat was used to calculate specific markers for each cluster compared with excluded cells, and only upregulated markers were reported. Minimum percentage of gene detection (min.pct) was set at 0.1 and threshold of log-scale difference (logFC) was set as 0.25. “FindNeighbors” and “FindClusters” functions in the Seurat package were used for clustering. We utilized the scCustomize R package [21] to facilitate visualization of Seurat. Additionally, we employed the Nebulosa package [22] to generate density maps that depicted the co-expression of genes, whilst Volcano plots were used to visualize differential gene expression between clusters.

### Scmap projection

Scmap (https://bioconductor.org/packages/release/bioc/html/scmap.html) is a tool for unsupervised projection between scRNAseq datasets [23]. We used Scmap to project cells from embryonic and adult cell populations to identify transcriptional similarity. Datasets for Scmap were constructed from the previous Seurat outputs.

### GO analysis

g:Profiler (https://biit.cs.ut.ee/gprofiler/gost) was utilized for Gene Ontology (GO) enrichment analysis to identify functional features of cluster-specific markers. The results of the GO enrichment analysis were exported and visualized with Graphpad Prism 9.0 (GraphPad Software, San Diego).

### Construction of PPI network

STRING (https://string-db.org/) is a biological database of known and predicted protein–protein interactions (PPI). We utilized STRING to construct a PPI network based on differentially expressed genes (DEGs), whilst the Cytoscape-based application CytoNCA (https://apps.cytoscape.org/apps/cytonca) scored protein interactions and selected the top 10 proteins for visualization [24].

### Reconstruction of cell differentiation trajectories

The Monocle3 R package (https://cole-trapnell-lab.github.io/monocle3/) was used to reconstruct cell differentiation trajectories [25]. First, the matrix data was extracted from previously defined Seurat objects to construct the cds file. We then used UMAP to reduce the dimensionality of the data and imported the integrated UMAP coordinates from Seurat. The “learn_graph()” function was used to construct the cell trajectories, then cells were ordered in pseudotime and visualized on UMAP. We employed the “graph_test()” function and utilized Moran’s index for spatial autocorrelation analysis to identify genes with differential expression across trajectories or clusters [26]. These genes were organized into modules according to their expression patterns and associated with respective clusters. Additionally, we utilized the “plot_genes_in_pseudotime()” function to visualize the dynamics of gene expression over pseudotime.

### SCENIC gene regulatory network inference analysis

Gene regulatory network analysis was performed using pySCENIC (v0.11.2, https://scenic.aertslab.org/) which is a lightning-fast python-based implementation of the SCENIC pipeline [27]. Seurat objects of cultured BMSCs and BMSC-derived neurospheres were converted to anndata objects through reticulate package and then written to loom files. Next, co-expression modules were inferred using a regression per-target approach (GRNBoost2). The cis-regulatory motif discovery (cisTarget) method was used to prune indirect targets from these modules. The activity of regulons was quantified by an enrichment score for the regulon’s target genes (AUCell). Regulon specificity scores (RSS) were calculated, and top 5 regulons from each cell cluster were selected. To visualize the activity of the regulons within different clusters, we imported the integrated loom file generated via SCENIC into the SCope visualization tool (http://scope.aertslab.org). We used the target genes of regulons identified by SCENIC as input objects to generate regulatory networks using iRegulon (http://iregulon.aertslab.org/) which is based on a combination of motif and epigenomic track annotations [28].

### Neurosphere differentiation

Day 8 BMSC-derived neurospheres were seeded onto poly-D-lysine / laminin-coated 4-well plates in neural differentiation medium comprising of neurobasal medium supplemented with 2% B27 and 1% GlutaMAX. For the SB4 treatment group, 1 μM SB4 was provided to bias differentiation. On day 10, cells were fixed for immunocytochemical analysis.

### Immunocytochemical analysis

Cells in culture were fixed with 4% PFA for 10 min at room temperature. Following PBS rinse, cells were incubated in blocking buffer comprising of PBS, 1% bovine serum albumin and 0.1% Triton × 100 for 30 min, followed by incubation with primary antibody at appropriate dilution in PBS overnight at 4 °C. After PBS rinse, cells were incubated with appropriate fluorochrome-conjugated secondary antibodies (1:400, Alexa Fluor®) for 1 h in the dark. Dapi stain was used to counterstain nuclei. Images were viewed under an inverted fluorescence microscope (Olympus BX53) and confocal microscope (Zeiss LSM880). Primary antibodies used in this study are listed in Additional file 1: Table S1.

### Quantitative real-time PCR assay

Total RNA of BMSC-derived neurospheres generated from three donors was extracted and purified using the RNeasy Kit (Qiagen, Düsseldorf, Germany) according to the manufacturer’s protocol. PrimeScript Reverse Transcriptase (Takara) with Oligo-(dt) primers were employed for complementary DNA (cDNA) synthesis. Quantitative real-time PCR was performed on Bio-Rad CFX96 Touch Real-Time PCR Detection System using SYBR Green Supermix (Bio-Rad). Primers used in this study are listed in Additional file 1: Table S2. The ratio of tested mRNA was normalized to GAPDH mRNA expression as the internal control using the formula: ΔCt = 2ˆ [Ct (target gene) − Ct (GAPDH)].

### Statistical analysis

Statistical analyses were performed using GraphPad Prism (v9.0, GraphPad Software). Data was presented as mean ± standard deviation. Student’s *t* test was used for statistical analysis, and *p*-values < 0.05 were considered statistically significant.

## Results

### Integrative analysis of single-cell transcriptome from human embryonic long bone to BMSC-derived neurospheres

**Fig. 1:**
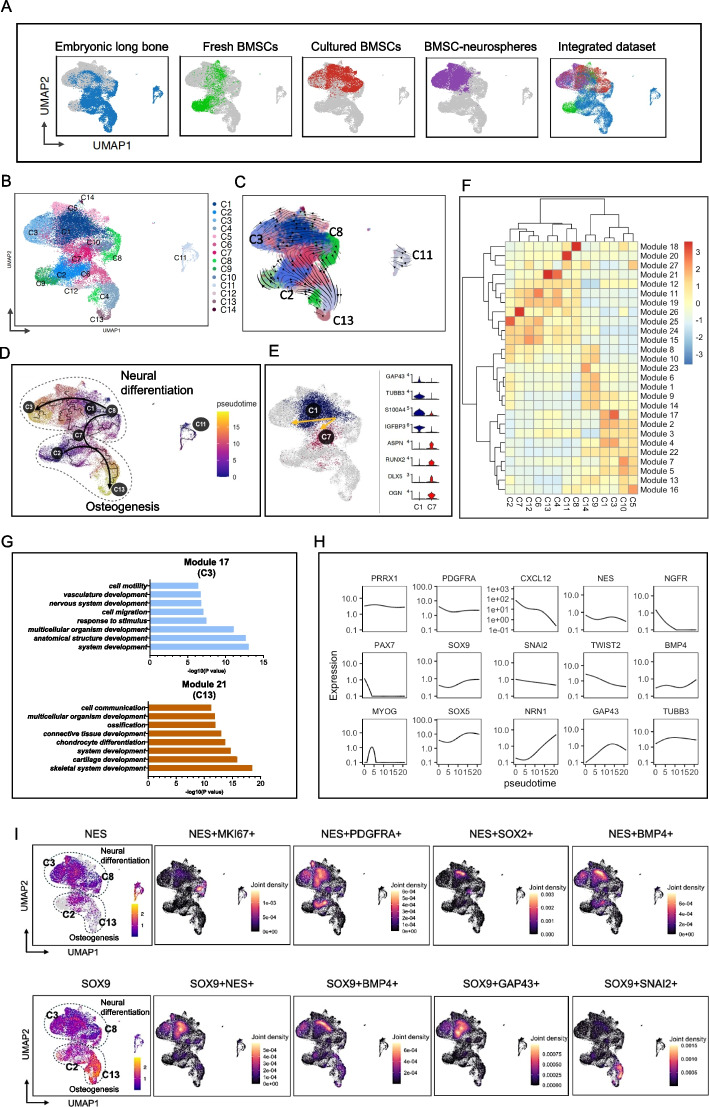
Integrated scRNAseq analysis of embryonic and adult bone marrow datasets. **A** UMAP characterizing the distribution of cells within each individual dataset (embryonic long bone, fresh BMSCs, cultured BMSCs, BMSC-neurospheres) and upon integration. **B** Unsupervised clustering of the integrated dataset yielded 14 clusters (C1-C14). **C** The RNA velocity graph revealed C8 and C2 as starting states, C3 and C13 as end points. **D** Pseudotime reconstruction indicated C3 (neural differentiation) and C13 (osteogenesis) were two distinct differentiation end states. **E** C1 and C7 were branch points originating from C8, with the former expressing genes associated with neural differentiation and the latter expressing genes related to skeletal development. **F** Identification of genes with differential expression across clusters was achieved via Moran’s index calculations. Subsequently, these genes were organized into 27 distinct modules based on their expression patterns, and associated with their corresponding clusters. **G** Functional enrichment analysis upon gene sets revealed that module 17 (correlating with C3) exhibited processes related to neurodevelopmental potential, whilst module 21 (correlating with C13) exhibited processes related to skeletal muscle development. **H** Along the pseudotime trajectory of the integrated dataset, a continuous decrease in the mesenchymal marker CXCL12 was detected. NC-related markers (P75, Pax7, Snail2 and Twist2) also decreased whilst on the contrary, neurogenesis-related genes (NRN1, GAP43 and TUBB3/TUJ1) increased. **I** Joint density map visualizing the co-expression pattern of genes in the integrated dataset

To better understand the transcriptomic characteristics of human BMSCs from embryo to adult as well as following in vitro expansion and neural induction, we integrated scRNAseq datasets of (i) human embryonic long bone and (ii) fresh (uncultured) adult human bone marrow aspirate, cultured human BMSCs and human BMSC-derived neurospheres for analysis (Fig. 1A). Sample details are further described in Additional file 1: Table S3. The greatest transcriptomic similarity was noted between cultured BMSCs and BMSC-derived neurospheres. Unsupervised clustering yielded 14 clusters from the integrated data (C1-C14, Fig. 1B), with differentially expressed genes for each integrated cluster and sample respectively shown in Additional file 2: Fig. S1A and B. Within the UMAP space, C11 was located away from other clusters and originated from the fresh BMSC sample. C11 robustly expressed neural crest stem cell markers Nestin/NGFR/ErbB3, and also expressed myogenic markers Pax7/myogenin/desmin (Additional file 2: Fig. S1C) whilst demonstrating biological processes related to muscle development and differentiation (Additional file 2: Fig. S1D, Additional file 1: Table S4). Taken together, we reasoned that this cluster resembled myogenic precursors that were descended from the neural crest [29, 30].

RNA velocity analysis (Fig. 1C) depicted C8 and C2 as starting points towards C3 and C13 clusters respectively. As detailed in Additional file 2: Fig. S1E, the differentiation branch from C8 to C3 was enriched in genes related to neural cell types (Nestin, Gap43, Tubb3, Nrn1) whilst the differentiation branch from C2 to C13 related to osteogenesis (Sox9/Col2A1/ACAN/SNORC). The C8 starting state corresponded to cells with a proliferative BMSC phenotype (see next section) which were found within the embryonic long bone sample. Following pseudotime reconstruction, we noted that C8 branched towards both C3 (neural differentiation) and C13 (osteogenesis) states, for the former by ways of a C1 intermediary cell state, and for the latter by ways of a C7 intermediary cell state (Fig. 1D). As shown in Fig. 1E, differentially expressed genes in C1 were associated with neural differentiation (GAP43, TUBB3, S100A4 and IGFBP3) whilst the C7 subpopulation exhibited high expression of genes related to skeletal development (ASPN, Runx2, Dlx5 and OGN). Moran’s index [26] was utilized to investigate differentially expressed genes between the 14 clusters within the integrated dataset, and we obtained 27 modules with similar gene expression patterns (Fig. 1F). With regard to end states, module 17 correlated with C3, and nervous system development was highlighted in functional enrichment analysis (Fig. 1G) whilst the hub gene was FN1 (Additional file 2: Fig. S1F). Module 21 correlated with C13, and biological processes related to ossification, connective tissue development and chondrocyte differentiation (Fig. 1G), whilst the hub gene was Sox9 (Additional file 2: Fig. S1F). With reference to the C7 branch point towards the C13 osteogenic end state, the hub gene was Col1A1 (Additional file 2: Fig. S1F), and biological processes related to skeletal system development (Additional file 2: Fig. S1G). Along the pseudotime trajectory of the integrated dataset (Fig. 1H), there was a continued decrease in NC-related markers (NGFR, Pax7, Snail2 and Twist2) and CXCL12 whilst on the contrary, neurogenesis-related genes (NRN1, GAP43 and TUBB3/TUJ1) increased.

Animal NC lineage tracing experiments [10] have demonstrated truncal NC to be the origin of Nestin + BMSCs within long bone marrow. We detected for cells with such a phenotype along the neurogenic differentiation branch originating from C8 (upper panels, Fig. 1I) which also expressed the proliferative marker MKI67 but exhibited reduced PDGFRA expression in comparison to the C2 starting state. Cells expressing Nestin/TUJ1 and GAP43/NRN1 characterized cells towards the mid- and end state of the neural differentiation trajectory (Additional file 2: Fig. S1H). Sox9 is important for both neural crest formation and skeletogenesis and was distributed along both differentiation pathways. In Sox9-expressing cells, co-expression of GAP43 was associated with the neural differentiation branch, whilst co-expression of Snail2 was associated with osteogenesis (Fig. 1I). The sample of origin for each subpopulation, as well as feature plots of each individual dataset are shown in Additional file 2: Fig. S1I and J respectively.

### Neural crest-like progenitors reside within human embryonic long bone and persist within adult bone marrow

We next characterized the transcriptional profile of human embryonic long bone harvested at up to 8 weeks post-conception [17] and identified 10 clusters (EC1-EC10, Additional file 2: Fig. S2A) which were phenotypically mature BMSCs (mBMSC, EC1), embryonic BMSCs (eBMSC, EC2), skeletal stem cells (SSC, EC4), proliferating BMSCs expressing MKI67/CDK1 (pBMSC, EC5), neural crest stem cells with a predilection for myogenesis corresponding to the C11 cluster upon integrated analysis (NCSC, EC7), Schwann cell precursors (SCP, EC10), pericytes (EC9), chondrocytes (EC8), chondroblasts (EC3) and osteoprogenitor cells (EC6). Markers utilized towards cellular identification are listed in Additional file 1: Table S5. The top 5 differentially expressed genes of each cluster were presented as a heat map (Additional file 2: Fig. S2B), the expression of neural and mesenchymal markers in relation to each cluster detailed upon a feature plot (Additional file 2: Fig. S2C), and the projections of each subpopulation onto the integrated dataset displayed in Additional file 2: Fig. S2D.

**Fig. 2:**
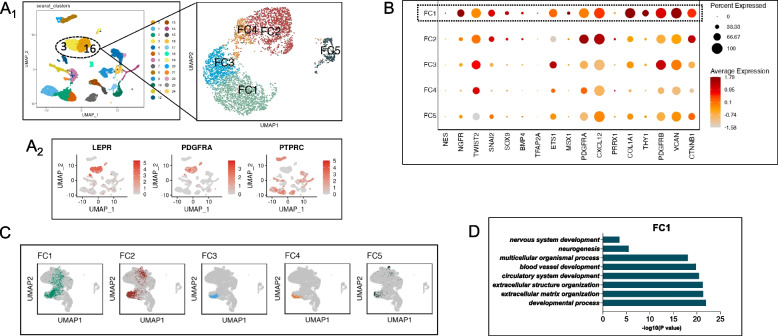
Neural crest-like progenitors are present within adult bone marrow. **A**_**1**_ UMAP visualization of 25 clusters identified in fresh adult human bone marrow, with BMSCs isolated in silico for re-clustering into 5 subpopulations (FC1-FC5) by using specific markers (LEPR^hi^/PDGFRA^hi^/ CD45^low^) which were concentrated at clusters 3 and 16 (**A**_**2**_). **B** Dot plot showing marker expression levels of each cluster within fresh BMSCs, with FC1 enriched in neural crest marker expression. **C** Projections of each subpopulation onto the integrated dataset indicated that FC1 was located amongst both neurogenic and osteogenic branches. **D** Results for biological process analysis, indicating FC1 possessed the potential for neural differentiation

We proceeded to identify NC-like progenitors within adult human bone marrow. From cells within the femoral head of adult patients, unsupervised clustering identified 25 distinct clusters (Fig. 2A1). Clusters 3 and 16 were juxtaposed upon UMAP analysis and were phenotypically BMSCs (LEPR^hi^/PDGFRA^hi^/ CD45^low^, Fig. 2A2) as has been defined previously [20]. We isolated these BMSCs in silico for re-dimensionalization and unsupervised clustering analysis. Amongst five distinct clusters (Fig. 2A1, B), FC1 resembled neural crest progenitors in demonstrating robust expression of NGFR and additionally the transcription factors Twist2/ETS1/MSX1 (Fig. 2B). Differentially expressed genes and feature plots of neural and mesenchymal marker expression are shown respectively in Additional file 2: Fig. S2E and F. Projection of FC1 upon the integrated dataset denoted the location of cells upon both the neural and osteogenesis regions of the integrated dataset whilst other clusters localized predominantly to the osteogenic region (Fig. 2C). Gene ontology (GO) analysis substantiated the functional capacity of FC1 towards nervous system development and neurogenesis (Fig. 2D). According to Scmap projection (Additional file 2: Fig. S2G), most cells (52.9%) from the FC1 subpopulation mapped to the mature BMSC (mBMSC) subpopulation.

### Characterization of BMSC-neurospheres reveals divergent differentiation of Nestin + /PDGFRA+ progenitors

**Fig. 3:**
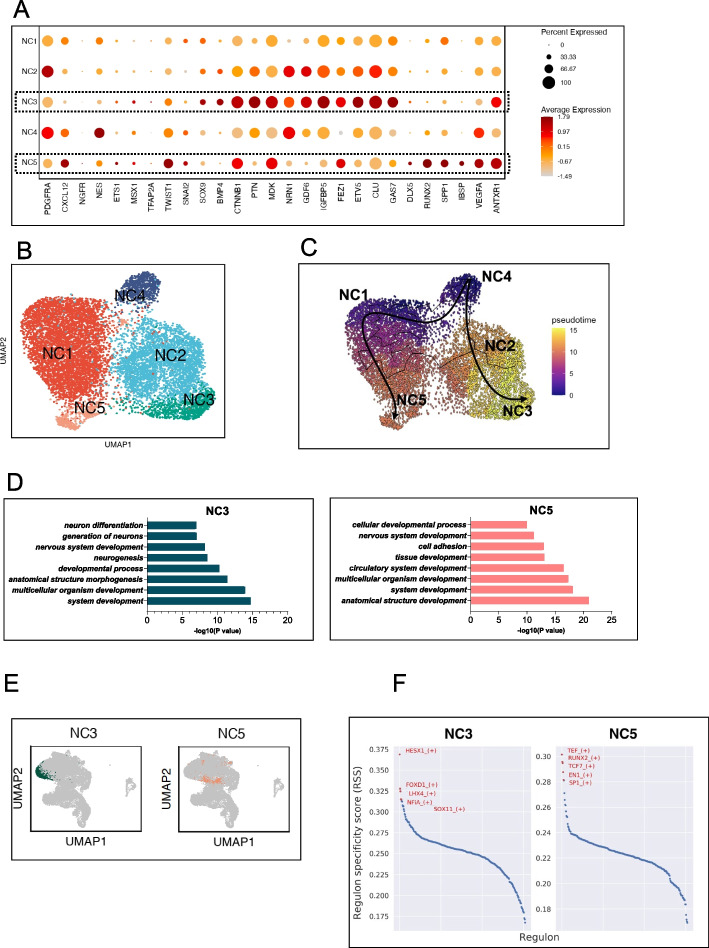
Characterization of subpopulations within BMSC-derived neurospheres. **A** Dot plot showing the marker expression levels of different clusters within BMSC-neurospheres. NC3 highly expressed CTNNB1, PTN, NRN1, GDF6, IGFBP5, FEZ1, ETV5, CLU and GAS7 which relate to neural development. NC5 highly expressed genes related to osteogenesis (DLX5, RUNX2, SPP1, IBSP) and vasculature formation (VEGFA, ANTXR1). **B** UMAP visualization of 5 clusters (NC1-NC5) identified within BMSC-neurospheres under unsupervised clustering. **C** The reconstructed pseudotime trajectory showed NC4 as the developmental starting state and NC3 and NC5 as the end states. **D** Functional enrichment analysis revealed that both NC3 and NC5 possessed the potential for neural differentiation. **E** Projections of NC3 and NC5 upon the integrated dataset. **F** The top 5 regulons in NC3 and NC5 clusters are highlighted in red, and regulon specificity scores (RSS) shown upon the *Y*-axis as inferred from the SCENIC algorithm

To gain insight into progenitor states within BMSC-neurospheres, unbiased clustering identified five clusters with distinct transcriptional profiles (NC1-NC5, Fig. 3A, B), with the Nestin + NC4 subpopulation being the starting point of differentiation according to the reconstructed pseudotime trajectory, which proceeded through NC1 and NC2 to reach the differentiation end states of NC5 and NC3 respectively (Fig. 3C). Differentially expressed genes in each cluster are shown in Additional file 2: Fig. S3A. GO analysis determined NC3 and NC5 to possess neural differentiation potential which distinguished them from other clusters (Fig. 3D, Additional file 2: Fig. S3B). Upon the integrated dataset, NC3 was situated at the terminal end of the neural differentiation branch, whereas NC5 was located at the bifurcation point towards osteogenesis. The location of other clusters upon UMAP is shown in Additional file 2: Fig. S3C. Whilst Nestin, PDGFRA and CTNNB1 were expressed by all clusters, CXCL12 expression was concentrated upon the trajectory towards NC5 whilst BMP4 and GDF6 were upregulated upon the end trajectory towards NC3 (Additional file 2: Fig. S3D). Comparison of differentially expressed genes in NC3 and NC5 are shown in Additional file 2: Fig. S3E. SCENIC revealed enrichment of transcription factors (Fig. 3G) in NC3 relating to neural development (HESX1 [31], LHX4 [32], NFIA [33]) and neural crest mesenchyme formation and maintenance (FOXD1 [34], SOX11 [35]). In NC5 on the other hand, SCENIC revealed transcription factors related to osteogenesis (Runx2 [36], TCF7 [37], EN1 [38] and SP1 [39]). By utilizing Scmap (Additional file 2: Fig. S3F), we identified common transcriptional states between the FC1 subpopulation of fresh BMSCs and each BMSC-neurosphere subpopulation. Similarity between the FC1 cluster and NC4 was highest (52.6%), the latter of which represented the starting neurosphere state in our preceding RNA velocity analysis.

### Directed differentiation of BMSC-derived neurospheres following transcriptomic analysis

**Fig. 4:**
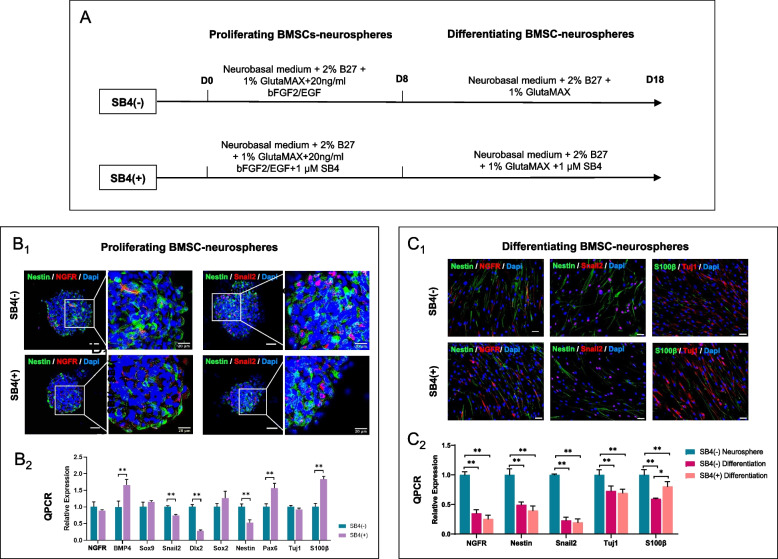
Culture of BMSC-neurospheres with BMP4 agonist. **A** Culture conditions for BMSC-neurosphere generation and differentiation, with and without provision of BMP4 agonist SB4. **B**_**1**_ Immunocytochemistry revealed that BMSC-derived neurospheres expressed neural crest marker NGFR and neural markers Nestin, Tuj1 and S100β. **B**_**2**_ Quantitative real-time PCR results revealed that SB4 treatment led to a significant upregulation in neural differentiation-related genes BMP4, Pax6 and S100β, and corresponding downregulation in mesenchymal-related gene Dlx2. **C**_**1**_ After 10 days of culture in neural differentiation medium, differentiating cells were spindle-shaped and expressed both neuronal (Tuj1) and glial (S100β) markers. **C**_**2**_ SB4 treatment during differentiation resulted in significantly higher expression of glial cell marker S100β. *N* = 3; **, *P* < 0.01; *, *P* < 0.05; Unlabelled scale bars: 50 μm

Analysis of BMSC-neurospheres had revealed an increase in BMP4 expression towards the neurogenic NC3 end state (Additional file 2: Fig. S3D). This was the rationale for treatment with SB4, a BMP4 signalling agonist, during expansion and differentiation of BMSC-neurospheres as detailed in Fig. 4A. Both treatment and control groups of BMSC-neurospheres demonstrated stochastic expression of the neural progenitor marker Nestin and neural crest markers NGFR / Snail2, with occasional colocalization (Fig. 4B1). S100β and Tuj1 (TUBB3) expression was also demonstrable. Amongst SB4-treated BMSC-derived neurospheres, upregulation of Pax6 coincided with Nestin downregulation (Fig. 4B2) which was taken as an indication of neuroectodermal fate bias of progenitor cells in response to culture conditions [40]. Downregulation of Dlx2 provided evidence of differentiation away from the mesenchymal fate [41]. Following passage and adherent culture in neural differentiation medium, cells adopted a tapered morphology (Fig. 4C1). Again, both treatment and control groups exhibited stochastic expression of the neural progenitor marker Nestin and neural crest markers NGFR/Snail2 with occasional colocalization. S100β and Tuj1 represented differentiation towards glial and neuronal fates respectively and were expressed in different cells. SB4-treated BMSC-neurospheres exhibited elevated S100β expression (Fig. 4C2), potentially indicating a glial predilection of differentiating neurospheres. Treatment groups did not differ in the expression of Nestin, NGFR/Snail2, or Tuj1 upon quantitative PCR.

## Discussion

The relevance of BMSCs for regenerative medicine has increased in recent years owing to growing evidence of genetic instability in iPSCs, such that clinical trials favour human embryonic stem cells as a progenitor source in spite of accompanying ethical concerns [3]. Analysing the transcriptomics of human BMSCs and their neural derivatives at single-cell resolution to delineate cellular identity and origin are essential prerequisites for application of these progenitors in cell therapy and disease modelling.

We sourced our bone marrow scRNAseq datasets from public repositories and obtained paired scRNAseq datasets of cultured BMSCs and BMSC-derived neurospheres from patients treated at our academic centre. Spatiotemporal variables considering, both cultured cells and bone marrow specimens originated from tibia and femur thus providing grounds for valid comparison. Upon integrative analysis, reconstructed pseudotime indicated cells to be distributed along both neural (C8 to C3) and osteogenic differentiation branches (C2 to C13). Culture of BMSC-neurospheres in bFGF/EGF was particularly effective in shifting the transcriptomic landscape towards the neural end state. Despite the C11 cluster being most neural crest-like, reconstructed pseudotime demonstrated C8 to be the starting state. C8 consisted of Nestin+ BMSCs with robust expression of mitotic markers and were bipotent via C1 and C7 branch points. Nestin+ BMSCs have been described to contain all bone marrow cells with colony-forming capacity [42]. Our results indicated that proliferating Nestin+ BMSCs gave rise to both mesenchymal and neural derivatives.

Few studies have been conducted on migration of human neural crest given the infeasibility of applying genetic tracing techniques [43]. Important transcriptional differences have been reported in comparison to mammalian counterparts in an immunohistochemical study conducted on aborted fetuses, for example in the dearth of HNK1 expression amongst migrating neural crest cells [44]. Culture conditions and passage number are known to significantly affect cellular phenotype [45], and the lack of NGFR in our cultured progenitor populations may be attributed to changes in the tissue niche as post-migratory NCSCs exhibit much more robust NGFR expression within the dorsal root ganglia as compared to long bone [9]. Nevertheless, Scmap demonstrated that a significant proportion of cells from BMSC-neurospheres exhibited similarity to the NGFR+ FC1 cluster amongst fresh BMSCs. Robust expression of Nestin / PDGFRA / CXCL12 amongst BMSC-neurospheres was compatible with cells originating from the truncal neural crest [10]. The dearth of PDGFRA expression at the C8 starting state as compared to robust expression at the C2 starting state was a pivotal difference. Amongst Nestin+ BMSCs, PDGFRA+ and PDGFRA− populations are segregated to respectively possess mesenchymal and glial differentiation capacity [10]. A gliogenic bias in the differentiation of BMSC-neuropheres is evidenced from our prior work [8]. Separately, Nestin+/CXCL12+ BMSCs comprise a distinct subpopulation that supports the haematopoietic stem cell niche [10]. With reference to these prior findings, our results are consistent with truncal neural crest as the origin of BMSCs with neural differentiation potential.

Differing from other scRNAseq studies characterizing human bone marrow [46], our major translational aim was to identify neural subpopulations within bone marrow for selection to improve upon the efficiency of differentiation. Integrated analysis identified Nestin + / MKI67 + BMSCs to possess both neural and mesodermal differentiation potential, whilst Sox9 + /Nestin + BMSCs corresponded to an intermediary state towards the neural end state along reconstructed pseudotime. FC1 (fresh BMSCs) and NC3 (BMSC-neurospheres) were also characterized to possess neural differentiation potential at these respective stages of cell isolation and culture. Proteomic analysis of these subpopulations would serve to identify discriminatory surface markers for cell selection. Additionally, our understanding on transcriptional dynamics identified signalling pathways at branch points to influence differentiation via defined culture conditions and in the absence of genetic manipulation. It was as a proof-of-principle that we provided SB4 to BMSC-neurospheres since a surge in BMP4 expression was observed towards the NC3 state. Neural crest induction requires intermediate levels of BMP4 negatively regulated by Noggin [47], in combination with Wnt/β-catenin and FGF pathway activity. When supplied to neural progenitors in vitro, BMP4 is a potent inducer of neuronal differentiation [48]. An increased expression of Pax6 and decreased expression of Nestin amongst SB4-treated neurospheres may have indicated differentiation towards a ventral neuronal progenitor phenotype [49]. BMP4 elevation after treatment also indicated a positive feedback loop, as has been demonstrated to be essential for CNS fate commitment of chordates [50].

Alternative peripheral sites from which multipotent progenitor cells with neural and mesenchymal potential may be harvested include the dental pulp [51] and skin [52]. An advantage in our progenitor source which was sourced from long bone marrow may be in the abundance of cells and relative accessibility. It is uncertain how the proliferation and neural differentiation potential of progenitor pools residing within other adult tissue niches compare, and this would warrant investigation.

In future work, BMSCs and BMSC-derived neurospheres from multiple patient donors should be characterized to confirm that similar subpopulations and transcriptional dynamics exist. Cell sorting of a neural crest-like population from freshly harvested bone marrow followed by clonal assays would demonstrate a single progenitor population possessing intrinsic neural and mesodermal differentiation potential and assuage the possibility of culture-induced transdifferentiation. Use of proliferation inhibitors would indicate that culture conditions expand upon this progenitor pool. To demonstrate cellular functionality, chimeric transplantation of bone marrow neural crest-like progenitors into the chick neural tube would be expected to result in migration to NC-specific tissues such as the dorsal root ganglia and dermis [53].

## Conclusions

BMSCs are posited as a promising progenitor source for autologous therapy given persistent concerns on the safety of iPSCs. Evidence from our scRNAseq analysis suggests that human BMSC-derived neural progenitors originated from the truncal neural crest, and specific subpopulations with neural differentiation potential have been identified. In preparation for translation, our findings provide critical insight for cell selection and efficient differentiation.

### Supplementary Information

**Additional file 1: Table S1.** Primary antibodies for immunofluorescence. **Table S2.** Primers for real-time quantitative (q) PCR. **Table S3.** Details of the scRNA-seq datasets. **Table S4.** Genes contributing to the GO Enrichment Analysis items. **Table S5.** Markers for assignment of cellular identity in embryonic long bone.

**Additional file 2: Fig. S1.** Integrative scRNAseq analysis of embryonic and adult bone marrow datasets. **Fig. S2.** Individual scRNAseq analysis of embryonic and adult bone marrow datasets. **Fig. S3.** Characterization of subpopulations within BMSC-derived neurospheres.

## Data Availability

The single-cell RNA sequencing datasets generated from our academic centre in this study are accessible at NCBI GEO website under the accession number GSE241499 (http://www.ncbi.nlm.nih.gov/geo/query/acc.cgi?acc=GSE241499).
