# Supplementary material for: Revealing the developmental origin and lineage predilection of neural progenitors within human bone marrow via single-cell analysis: implications for regenerative medicine
Source: Genome Med. 2023 Sep 4;15:66. doi: 10.1186/s13073-023-01224-0 (PMC10476295; doi:10.1186/s13073-023-01224-0)
Supplement: Supplementary file 1 — Additional file 1: Table S1. Primary antibodies for immunofluorescence. Table S2. Primers for real-time quantitative (q) PCR. Table S3. Details of the scRNA-seq datasets. Table S4. Genes contributing to the GO Enrichment Analysis items. Table S5. Markers for assignment of cellular identity in embryonic long bone. [file 13073_2023_1224_MOESM1_ESM.pdf]

Table S1: Primary antibodies for immunofluorescence

Antibody	Concentration	Manufacturer
Nestin (Mouse IgG)	1:200	BD Pharmingen
NGFR (Rabbit IgG)	1:1000	Abcam
Snail2 (Rabbit IgG)	1:200	Cell Signaling Technology
S100β(Mouse IgG)	1:100	Abcam
Tuj1 (Rabbit IgG)	1:500	BioLegend

Table S2: Primers for real-time quantitative (q) PCR

Markers	Forward primer	Reverse primer
NGFR	GGCACCGCCTTCTCTAAATG	GCAAGCATCCCATCTCCAC
BMP4	AAGAGCAGATCCACAGCACT	AAGCAGAGTTTTCACTGGTCC
Sox9	TGCAGGAGGAGAAGAGAAGG	GTGGCCAGTTCACAGCTGC
Snail2	CTGGTCAAGAAGCATTTCAACG	AGGATCTCTGGTTGTGGTATGA
Dlx2	ATGGGTTCCTACCAGTACCAA	TCCTTCTCAGGCTCGTTGTT
Sox2	GAGCTTTGCAGGAAGTTTGC	GCAAGAAGCCTCTCCTTGAA
Nestin	AACAGCGACGGAGGTCTCTA	TTCTCTTGTCCCGCAGACTT
Pax6	AGGGCCAAATGGAGAAGAGAA	GGTAGACACTGGTGCTGAAAC
Tuj1	GCGCATCAGCGTATACTACAA	TTCCAAGTCCACCAGAATGG
S100β	TCATCAACAATGAGCTTTCCCA	ATTCGCCGTCTCCATCATTGT
GAPDH	GACGCTGGGGCTGGCATTG	GCTGGTGGTCCAGGGGTC

Table S3: Details of the scRNA-seq datasets

Name	Sample size (n)	Age (sex)	Origin	GEO number
Human embryonic long bones	3	8 weeks post conception	Embryonic long bones	GSE143753
Fresh BMSC	2	84-year-old (male)<br>67-year-old (female)	Femoral head bone marrow aspirates	GSE147287
Cultured BMSC	1	41-year-old (female)	Long bone aspirates	/
BMSC-neurosphere	1	41-year-old (female)	Long bone aspirates	/

Table S4: Genes contributing to the GO Enrichment Analysis items

Cluster	Biological process items	Intersections
C11	muscle structure development	TNNI1,MYOG,PROX1,ACTN2,RYR2,ZBTB18,NEB,TANC1,XIRP2,KLHL41,CHRNA1,TTN,MSTN,DES,PAX3,CHRND,CAV3,TNNC1,LMOD3,MYOZ2,PDLIM3,MEGF10,RBM24,MYMX,COL19A1,VGLL2,DLL1,LMOD2,MSC,JPH1,HEY1,ADGRB1,TMOD1,CAVIN4,MYOZ1,SYNPO2L,LDB3,ANKRD1,SMTNL1,HSPB2,ITGA7,ERBB3,STAC3,MYF6,FGF9,AKAP6,ASB2,ACTC1,ALPK3,TRIM72,CHRNB1,MYH3,TCAP,SGCA,RNF165,NMRK2,RYR1,SIRT2,DMPK,MAMSTR,TNNT1,TNNI3,JPH2,RBM38,MYO18B,FOXO4,ITGB1BP2,SRPK3,PAX7,RXRG,ACTA1,SMYD1,LBX1,BARX2,MYF5,PDGFB,POU4F1,UNC45B,CACNA1S,TNNT2,KLHL40,MYMK,MYOD1
	muscle cell differentiation	MYOG,PROX1,ACTN2,NEB,TANC1,KLHL41,TTN,CAV3,LMOD3,MYOZ2,MEGF10,RBM24,MYMX,DLL1,LMOD2,HEY1,ADGRB1,TMOD1,MYOZ1,SYNPO2L,LDB3,ANKRD1,STAC3,MYF6,FGF9,AKAP6,ASB2,ACTC1,ALPK3,TRIM72,CHRNB1,MYH3,TCAP,RYR1,DMPK,MAMSTR,TNNT1,RBM38,MYO18B,FOXO4,ACTA1,SMYD1,BARX2,MYF5,PDGFB,CACNA1S,TNNT2,KLHL40,MYMK,MYOD1
FC1	neurogenesis	MDK,THY1,VCAN,FZD1,CLU,NLGN4X,FLNA,APOE,CDH11,EFNB1,NGFR,UBB,FN1,PLXNB2,ANXA2,GRN,MARCKS,COL3A1,PRMT1,UNC5B,MMP2,IL34,VIM,SLIT3,TNC,DDIT4,CALR,ADM,GAS6,HSPA5,GPM6B,ID4,ID3,PLEC,EDNRA,ENAH,HES1,MME,NDRG1,ARF4,NOTCH3,HSP90AA1,JUN,CNTNAP2,ARHGEF10,NAV3,VCL,RTN4RL1,SERPINF1,SOX4,RPL24,LRP1,HGF,UGCG,MXRA8,LAMB1,DCLK1,SLC1A3,PALLD,GLDN
	nervous system development	MDK,THY1,VCAN,FZD1,CLU,NLGN4X,FLNA,APOE,CDH11,EFNB1,NGFR,UBB,FN1,PLXNB2,ANXA2,DLC1,GRN,MARCKS,COL3A1,PRMT1,UNC5B,COL4A1,MMP2,IL34,VIM,SLIT3,TNC,DDIT4,APOB,CALR,ADM,GAS6,GRIK1,HSPA5,DYNLL1,CALM3,GPM6B,ID4,ID3,PLEC,EDNRA,ENAH,HES1,MME,NDRG1,PLCB1,HSPG2,CTSC,ARF4,NOTCH3,CYP1B1,HSP90AA1,JUN,CNTNAP2,FOXC1,ARHGEF10,NAV3,MTHFD1L,VCL,RTN4RL1,SERPINF1,SOX4,RPL24,LRP1,HGF,UGCG,MXRA8,LAMB1,ZEB2,DCLK1,SLC1A3,PALLD,GLDN
NC3	neurogenesis	HHIP,CLU,PTPRD,PTN,FOXD1,GPM6B,GDF6,ITM2C,CTNNB1,TENM4,ID1,CDKN1C,LRP1,BMP4,PTK2,ID3,TCF4,PPP3CA,NRN1,GBA2,GAS7,EPHB2,PLXNA2,MDK,ALCAM,FEZ1,S100A10,TMEM98,PBX1,SOX4,PTPRG,RAB11A,NTN1,ZNF521,ZEB1,STMN1,APOD,MEF2C,MAN2A1,FAT3
	nervous system development	HAPLN1,HHIP,CLU,PTPRD,PTN,FOXD1,GPM6B,GDF6,ITM2C,CTNNB1,TENM4,ID1,MAFB,CDKN1C,MARCKSL1,LRP1,BMP4,PTK2,PITX1,ID3,TCF4,PPP3CA,NRN1,GBA2,GAS7,EPHB2,PLXNA2,MDK,ALCAM,FEZ1,S100A10,TMEM98,PBX1,SOX4,NFIA,STK3,PTPRG,RAB11A,NTN1,ZNF521,ECRG4,ZEB1,NINJ1,STMN1,APOD,MEF2C,SSBP3,MAN2A1,FAT3
NC5	nervous system development	SERPINF1,TENM4,CADM1,SLIT2,CTHRC1,PPP3CA,CTSC,THBS2,GSN,MARCKSL1,PALLD,TWIST1,S100A10,CHRM3,MARCKS,PAM,ETV1,PTPRD,PTPN13,SATB2,NAV3,SYNJ2,ITGA1,HGF,CMTM8,HDAC9,NREP,MAFB,SOX4,CXCL12,SLC1A3,CTNNB1,CEBPB,NR2F2,BHLHE41,IRX3,TGFBR2,IGF2BP2,FEZ1,B4GALT5,DOCK10,SERINC5,JUN,SPOCK1,ZFP36L1,NEO1,RYK,VEGFA,PRRX1,ETV6,TBX2,BEND6,NTM,GPC4,TGM2,SRGAP2B,BASP1,TUBB3,MEIS2,WNT5B,TGFB2,TANC2,ZNF521,SPP1

Table S5: Markers for assignment of cellular identity in embryonic long bone

Cell type	Markers
mBMSC	PDGFRA^{high+}/Cxcl12^{high+}
eMSC	PDGFRA+ / Cxcl12+ / Pitx1+ / Foxp1+ / GAS1+ / Twist1+ / Twist2+
Osteoprogenitor	OGN+/ Prrx1+/Runx2+/Dlx5+
pBMSC	PDGFRA+ / Cxcl12+ / MKI67+ / CDK1+
Chondroblast	Sox9+ / COL2A1+
Chondrocyte	Sox9+ / COL2A1 $^{high+}$/ SNORC $^{high+}$/ ACAN $^{high+}$
SSC	SFRP2+ / OSR2+ / CDON+ / Pitx1+/ Foxp1+ / GAS1+
Pericyte	MCAM+ / PDGFRB+
NCSC	MYC+ / NES+ / PAX7+ / SOX8+ / NGFR+ / Snail2+
SCP	NGFR+ / S100B+ / ERBB3+ / Sox10+ / Plp1+ / P0+
