# Supplementary material for: Revealing the developmental origin and lineage predilection of neural progenitors within human bone marrow via single-cell analysis: implications for regenerative medicine
Source: Genome Med. 2023 Sep 4;15:66. doi: 10.1186/s13073-023-01224-0 (PMC10476295; doi:10.1186/s13073-023-01224-0)
Supplement: Supplementary file 2 — Additional file 2: Fig. S1. Integrative scRNAseq analysis of embryonic and adult bone marrow datasets. Fig. S2. Individual scRNAseq analysis of embryonic and adult bone marrow datasets. Fig. S3. Characterization of subpopulations within BMSC-derived neurospheres. [file 13073_2023_1224_MOESM2_ESM.pdf]

Fig. S1

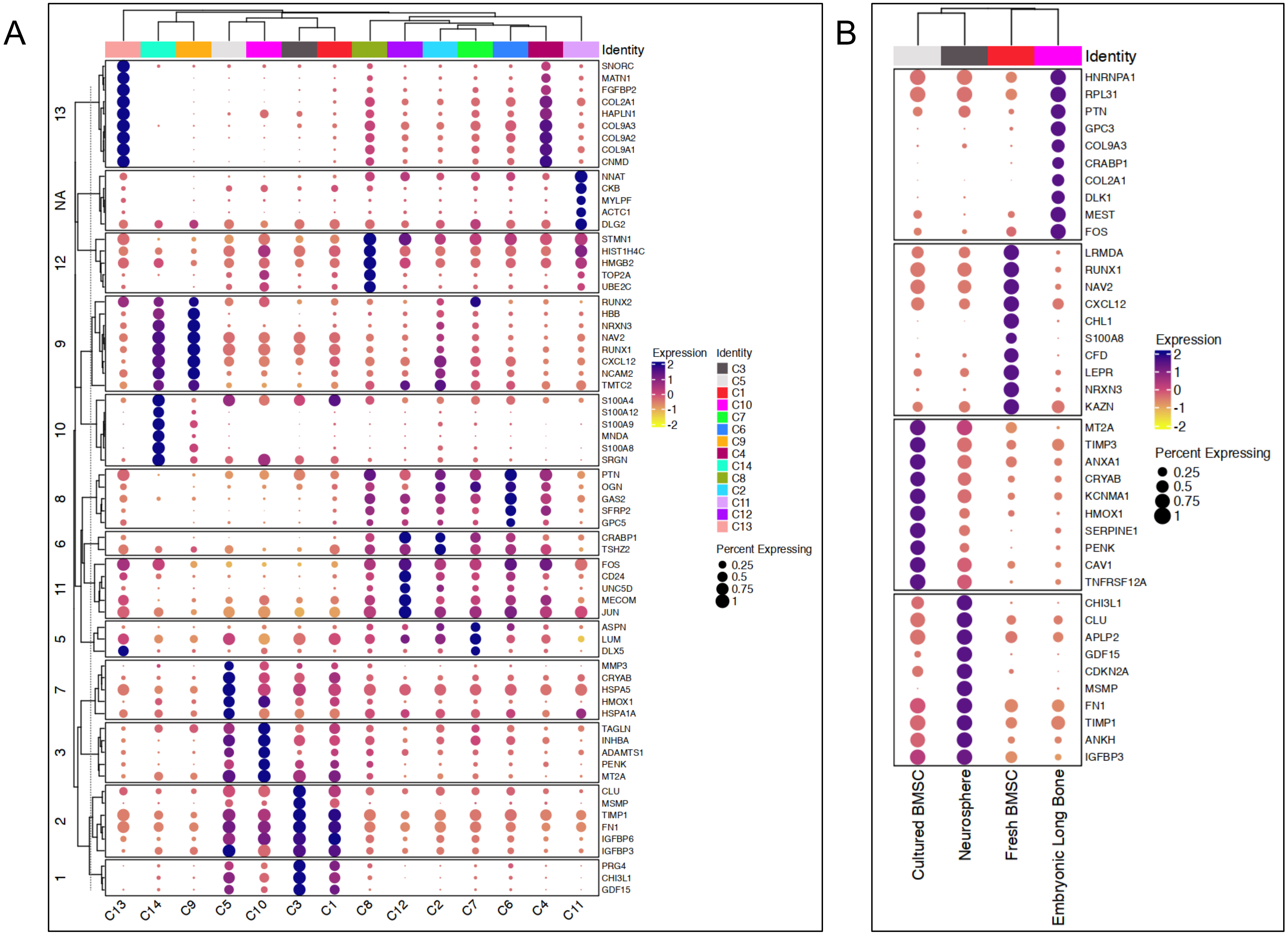
A
Identity
SNORC
MATN1
FGFBP2
COL2A1
HAPLN1
COL9A3
COL9A2
COL9A1
CNMD
NNAT
CKB
MYLPF
ACTC1
DLG2
STMN1
HIST1H4C
HMGB2
TOP2A
UBE2C
RUNX2
HBB
NRXN3
NAV2
RUNX1
CXCL12
NCAM2
TMTC2
S100A4
S100A12
S100A9
MNDA
S100A8
SRGN
PTN
OGN
GAS2
SFRP2
GPC5
CRABP1
TSHZ2
FOS
CD24
UNC5D
MECOM
JUN
ASPN
LUM
DLX5
MMP3
CRYAB
HSPA5
HMOX1
HSPA1A
TAGLN
INHBA
ADAMTS1
PENK
MT2A
CLU
MSMP
TIMP1
FN1
IGFBP6
IGFBP3
PRG4
CHI3L1
GDF15
13
NA
12
9
10
8
6
11
5
7
3
2
1
C13
C14
C9
C5
C10
C3
C1
C8
C12
C2
C7
C6
C4
C11
Expression
2
1
0
-1
-2
Identity
C3
C5
C1
C10
C7
C6
C9
C4
C14
C8
C2
C11
C12
C13
Percent Expressing
0.25
0.5
0.75
1
B
Identity
HNRNPA1
RPL31
PTN
GPC3
COL9A3
CRABP1
COL2A1
DLK1
MEST
FOS
LRMDA
RUNX1
NAV2
CXCL12
CHL1
S100A8
CFD
LEPR
NRXN3
KAZN
MT2A
TIMP3
ANXA1
CRYAB
KCNMA1
HMOX1
SERPINE1
PENK
CAV1
TNFRSF12A
CHI3L1
CLU
APLP2
GDF15
CDKN2A
MSMP
FN1
TIMP1
ANKH
IGFBP3
Cultured BMSC
Neurosphere
Fresh BMSC
Embryonic Long Bone
Expression
2
1
0
-1
-2
Percent Expressing
0.25
0.5
0.75
1

Fig. S1

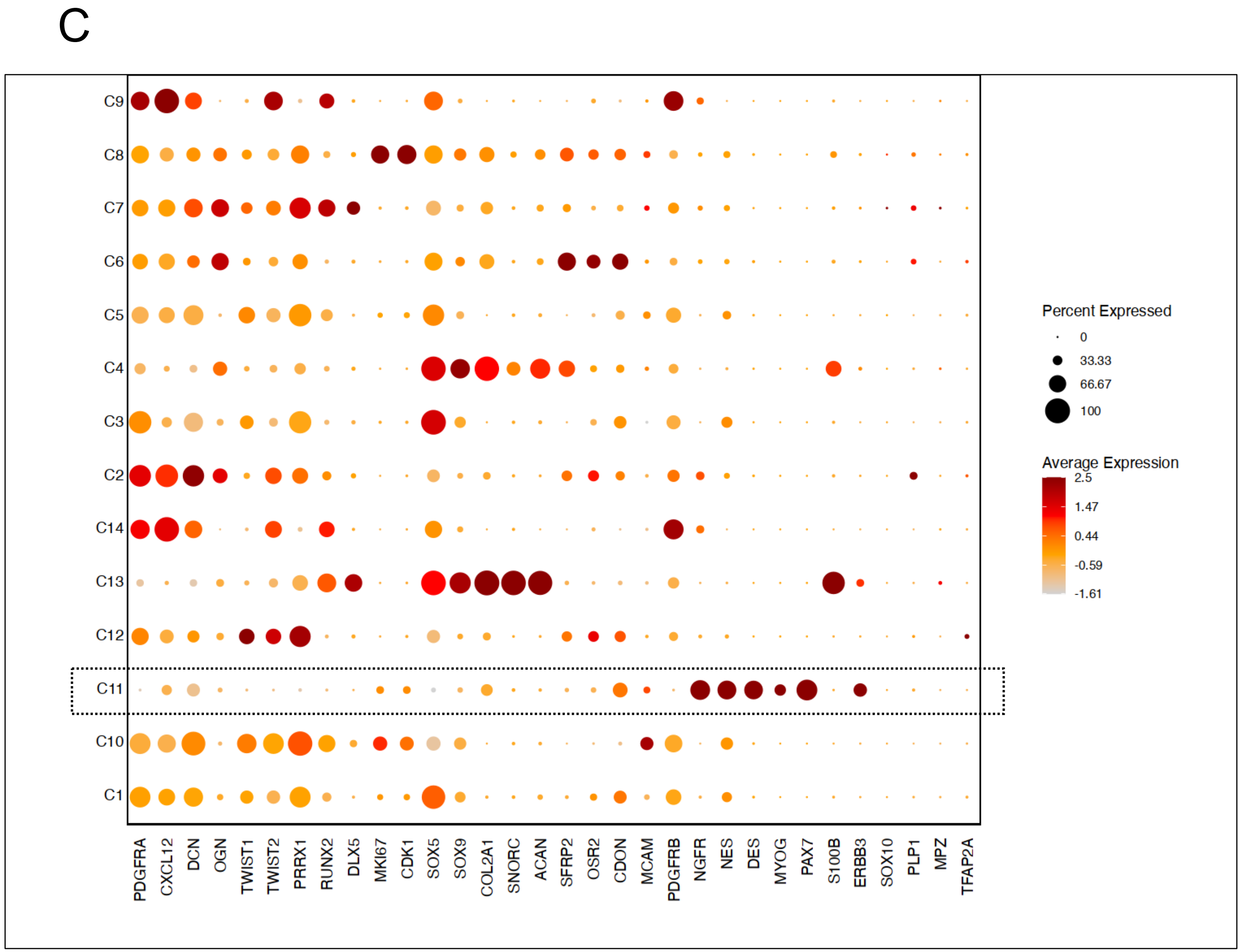

C
C9
C8
C7
C6
C5
C4
C3
C2
C14
C13
C12
C11
C10
C1
PDGFRA
CXCL12
DCN
OGN
TWIST1
TWIST2
PRRX1
RUNX2
DLX5
MKI67
CDK1
SOX5
SOX9
COL2A1
SNORC
ACAN
SFRP2
OSR2
CDON
MCAM
PDGFRB
NGFR
NES
DES
MYOG
PAX7
S100B
ERBB3
SOX10
PLP1
MPZ
TFAP2A
Percent Expressed
0
33.33
66.67
100
Average Expression
2.5
1.47
0.44
-0.59
-1.61

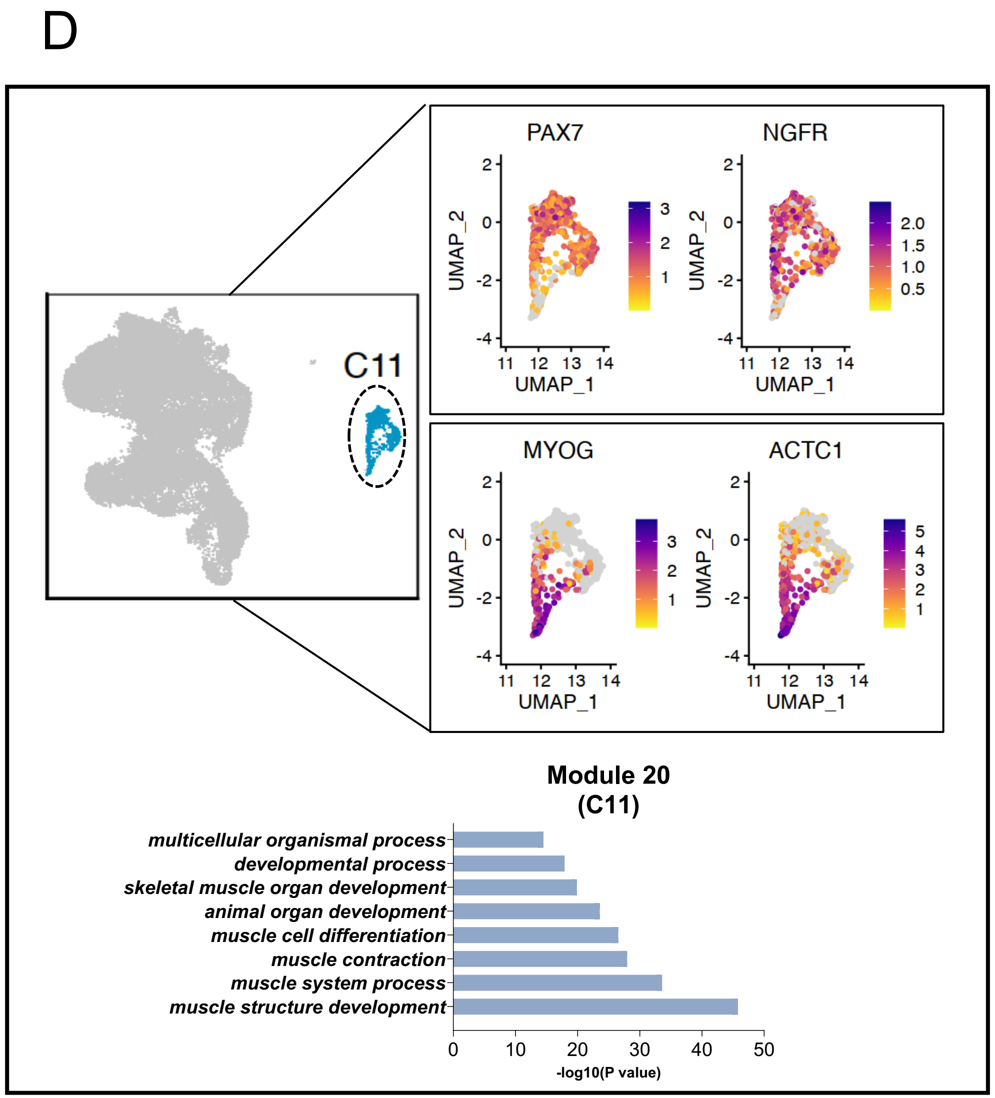

D
C11
PAX7
NGFR
MYOG
ACTC1
UMAP_2
UMAP_1
Module 20
(C11)
multicellular organismal process
developmental process
skeletal muscle organ development
animal organ development
muscle cell differentiation
muscle contraction
muscle system process
muscle structure development
-log10(P value)

Fig. S1

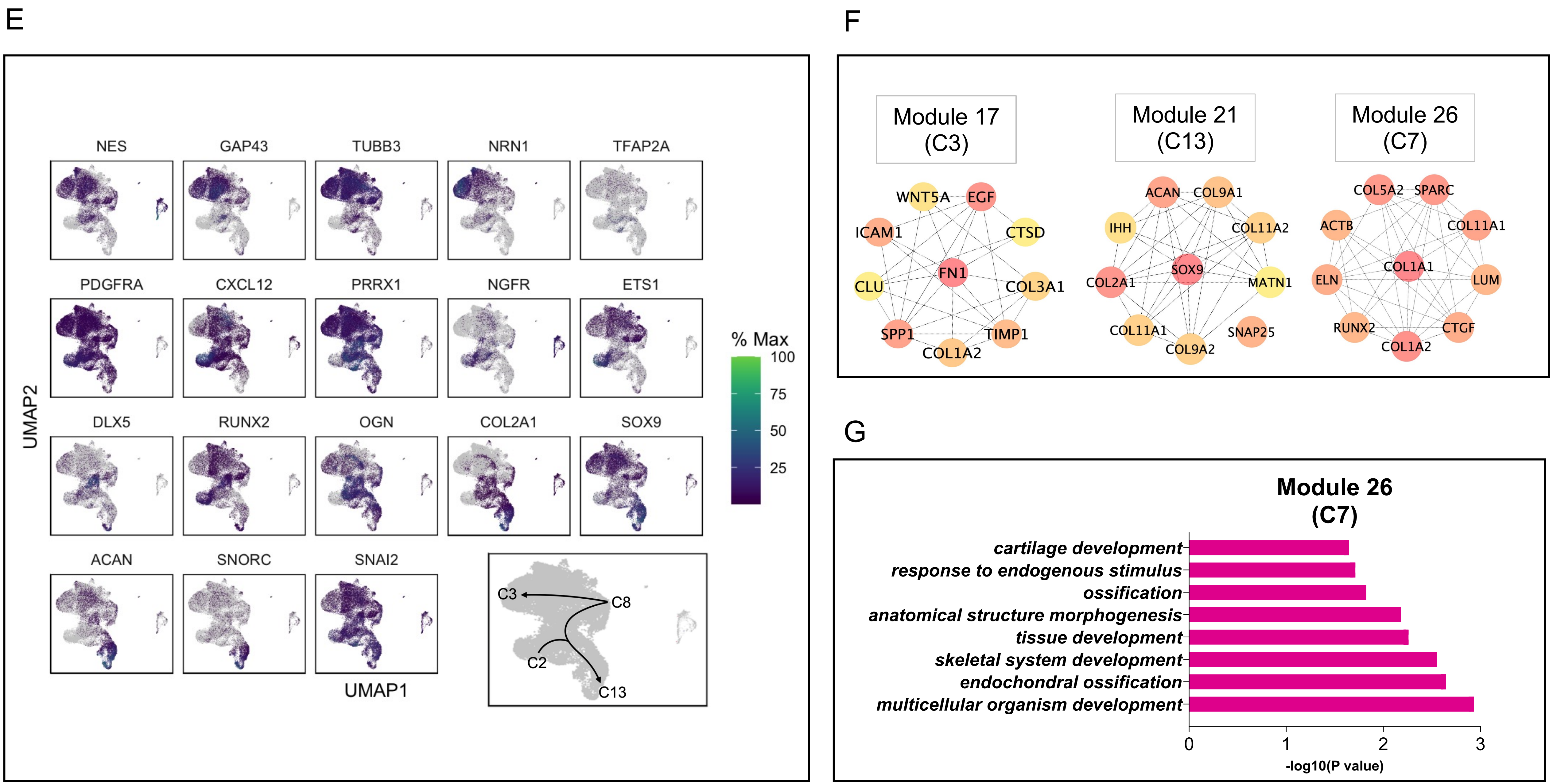
E
NES
GAP43
TUBB3
NRN1
TFAP2A
PDGFRA
CXCL12
PRRX1
NGFR
ETS1
DLX5
RUNX2
OGN
COL2A1
SOX9
ACAN
SNORC
SNAI2
UMAP2
UMAP1
% Max
100
75
50
25
C3
C8
C2
C13
F
Module 17 (C3)
WNT5A
EGF
ICAM1
CTSD
FN1
CLU
COL3A1
SPP1
TIMP1
COL1A2
Module 21 (C13)
ACAN
COL9A1
IHH
COL11A2
SOX9
COL2A1
MATN1
COL11A1
SNAP25
COL9A2
Module 26 (C7)
COL5A2
SPARC
ACTB
COL11A1
COL1A1
ELN
LUM
RUNX2
CTGF
COL1A2
G
Module 26 (C7)
cartilage development
response to endogenous stimulus
ossification
anatomical structure morphogenesis
tissue development
skeletal system development
endochondral ossification
multicellular organism development
0
1
2
3
-log10(P value)

Fig. S1

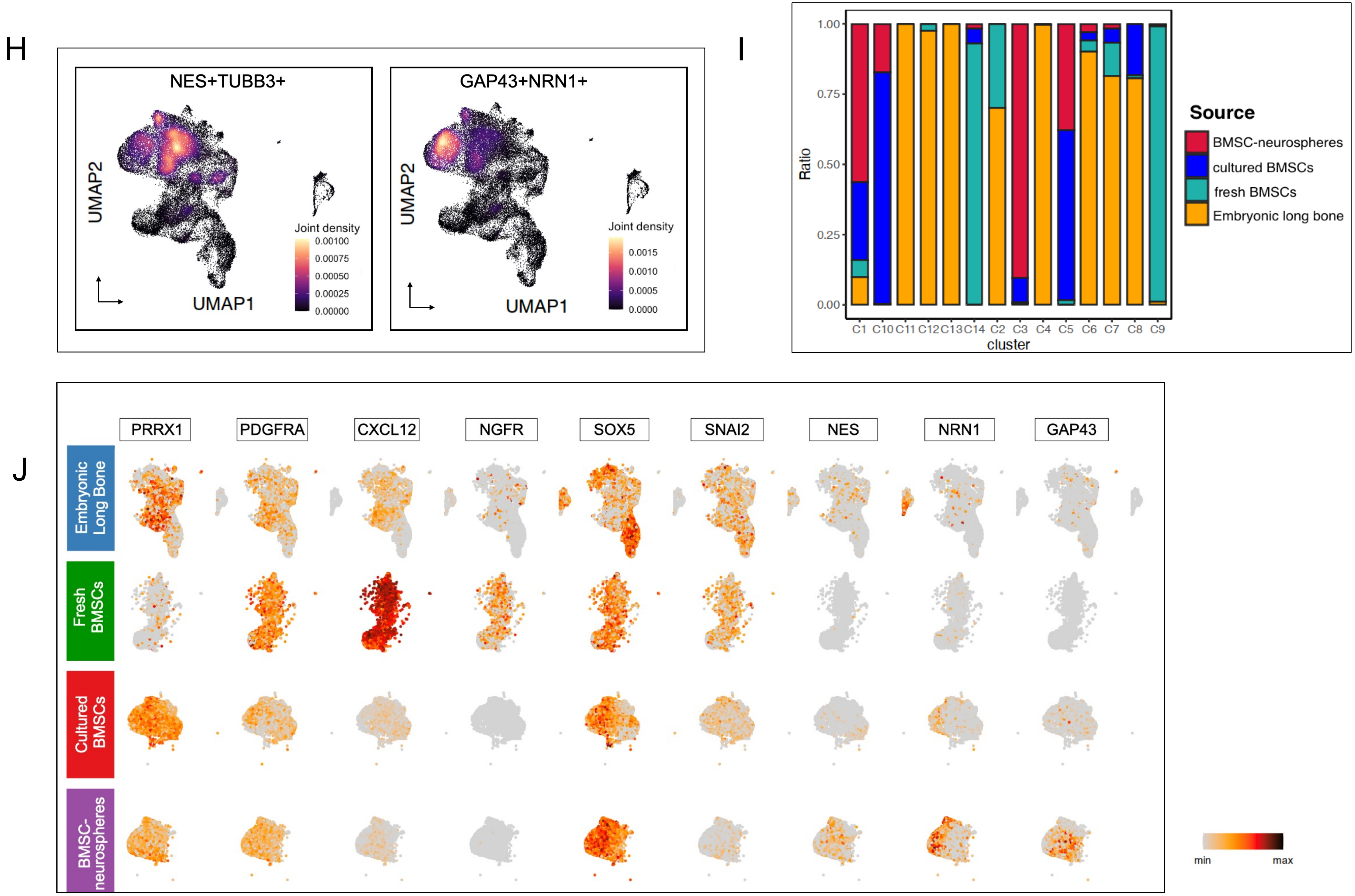
H
NES+TUBB3+
GAP43+NRN1+
UMAP2
UMAP1
Joint density
0.00100
0.00075
0.00050
0.00025
0.00000
0.0015
0.0010
0.0005
0.0000
I
Ratio
1.00
0.75
0.50
0.25
0.00
C1 C10 C11 C12 C13 C14 C2 C3 C4 C5 C6 C7 C8 C9
cluster
Source
BMSC-neurospheres
cultured BMSCs
fresh BMSCs
Embryonic long bone
J
PRRX1
PDGFRA
CXCL12
NGFR
SOX5
SNAI2
NES
NRN1
GAP43
Embryonic Long Bone
Fresh BMSCs
Cultured BMSCs
BMSC-neurospheres
min
max

Fig. S1

(A) Dot plot showing top 5 differentially expressed genes from 14 distinct clusters amongst integrated dataset as well as (B) top 10 differentially expressed genes for each individual dataset which included embryonic long bone, cultured BMSCs, fresh BMSCs and BMSC-neurospheres. (C) Dot plot showing the marker expression levels of different clusters within the integrated dataset. (D) Analysis of the C11 subpopulation revealed high expression of neural crest markers Pax7 and NGFR in the early stages, followed by a shift towards myogenic markers MYOG and ACTC1 in the later stages, suggesting a potential for myogenic differentiation which was supported by biological process analysis. (E) Expression of neural and osteogenic markers upon the integrated UMAP. (F) PPI analysis revealed that the hub gene in module 17 (corresponding to C3) was FN1, the hub gene of module 21 (corresponding to C13) was Sox9, while the hub gene of module 26 (corresponding to C7) was Col1A1. (G) Results for biological process analysis, indicating C7 possessed the potential for skeletal system development. (H) Joint density map showing the co-expression of Nestin/TUBB3, and GAP43/NRN1 along the neural differentiation branch. (I) Stacked bar chart revealing the composition of the cells within each cluster. (J) Feature plot of each individual dataset showing the expression of mesenchymal, neural crest, and neuronal markers.

Fig. S2

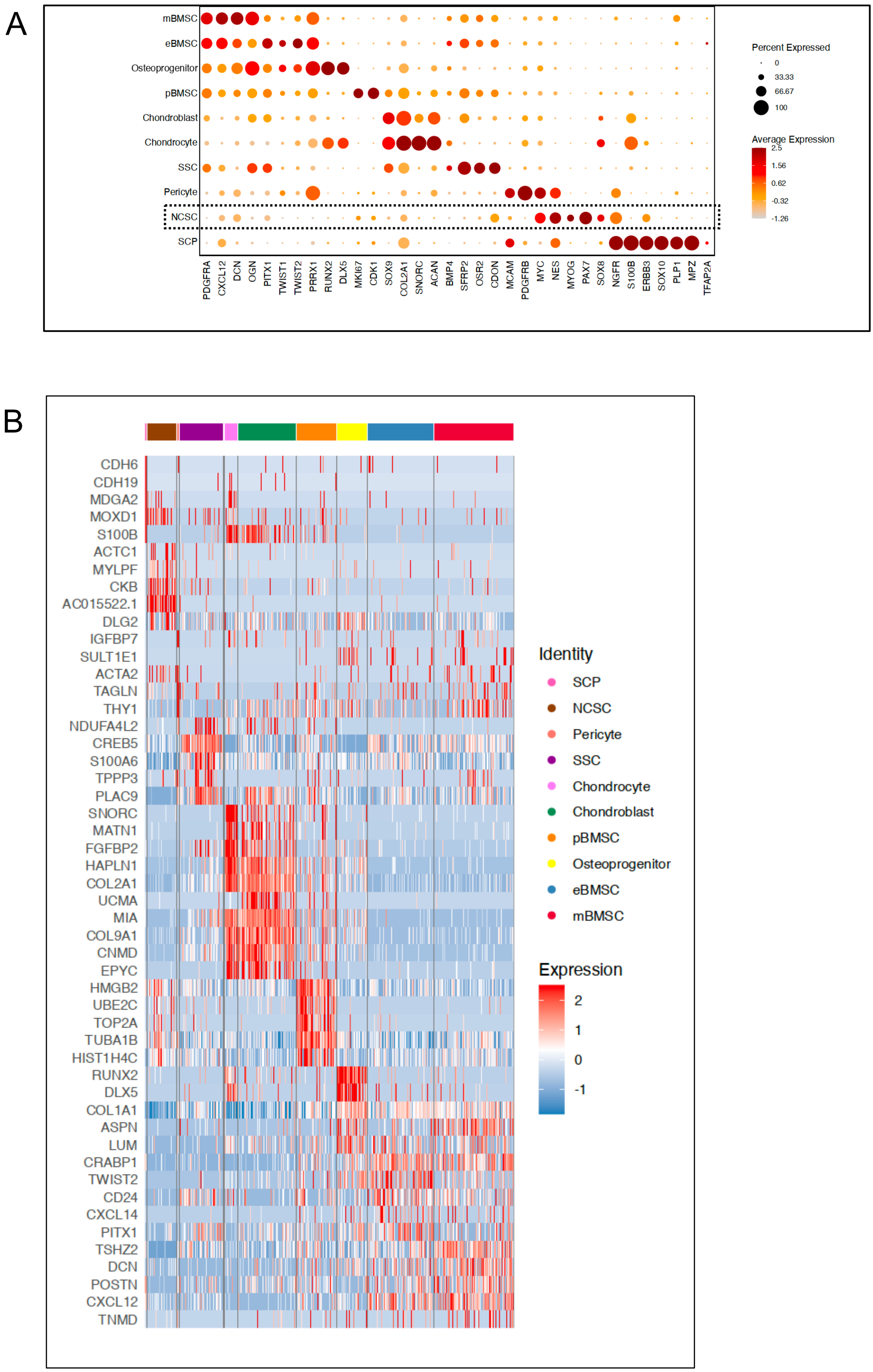
A
mBMSC
eBMSC
Osteoprogenitor
pBMSC
Chondroblast
Chondrocyte
SSC
Pericyte
NCSC
SCP
PDGFRA CXCL12 DCN OGN PITX1 TWIST1 TWIST2 PRRX1 RUNX2 DLX5 MKI67 CDK1 SOX9 COL2A1 SNORC ACAN BMP4 SFRP2 OSR2 CDON MCAM PDGFRB MYC NES MYOG PAX7 SOX8 NGFR S100B ERBB3 SOX10 PLP1 MPZ TFAP2A
Percent Expressed
0
33.33
66.67
100
Average Expression
2.5
1.56
0.62
-0.32
-1.26
B
CDH6
CDH19
MDGA2
MOXD1
S100B
ACTC1
MYLPF
CKB
AC015522.1
DLG2
IGFBP7
SULT1E1
ACTA2
TAGLN
THY1
NDUFA4L2
CREB5
S100A6
TPPP3
PLAC9
SNORC
MATN1
FGFBP2
HAPLN1
COL2A1
UCMA
MIA
COL9A1
CNMD
EPYC
HMGB2
UBE2C
TOP2A
TUBA1B
HIST1H4C
RUNX2
DLX5
COL1A1
ASPN
LUM
CRABP1
TWIST2
CD24
CXCL14
PITX1
TSHZ2
DCN
POSTN
CXCL12
TNMD
Identity
SCP
NCSC
Pericyte
SSC
Chondrocyte
Chondroblast
pBMSC
Osteoprogenitor
eBMSC
mBMSC
Expression
2
1
0
-1

Fig. S2

C

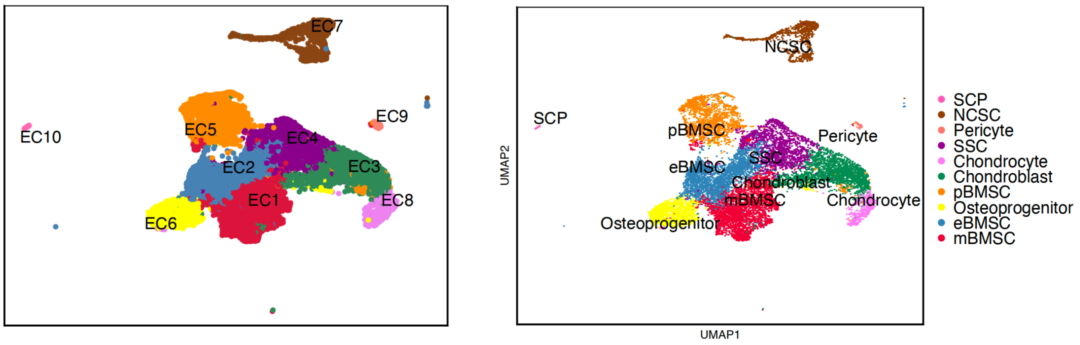
EC7
EC10
EC5
EC9
EC4
EC2
EC3
EC1
EC8
EC6
NCSC
SCP
pBMSC
Pericyte
eBMSC
SSC
Chondroblast
mBMSC
Chondrocyte
Osteoprogenitor
UMAP2
UMAP1
SCP
NCSC
Pericyte
SSC
Chondrocyte
Chondroblast
pBMSC
Osteoprogenitor
eBMSC
mBMSC

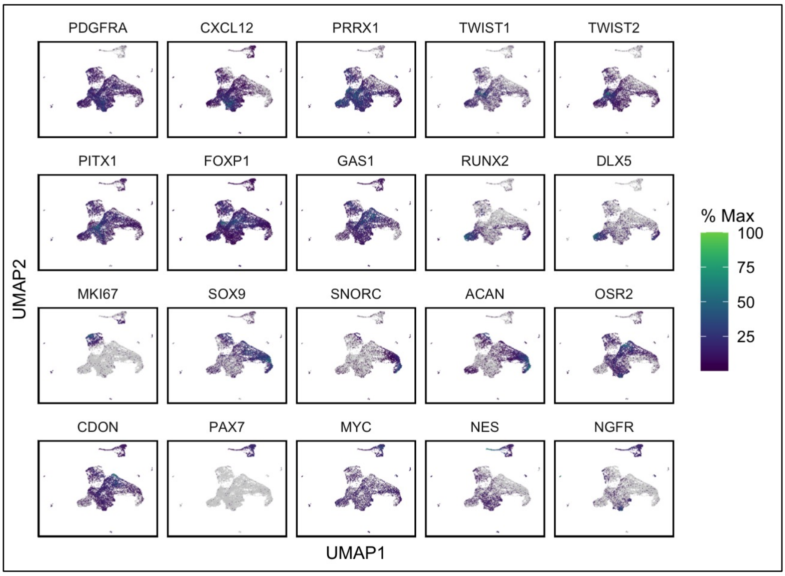
PDGFRA
CXCL12
PRRX1
TWIST1
TWIST2
PITX1
FOXP1
GAS1
RUNX2
DLX5
MKI67
SOX9
SNORC
ACAN
OSR2
CDON
PAX7
MYC
NES
NGFR
UMAP2
UMAP1
% Max
100
75
50
25

D

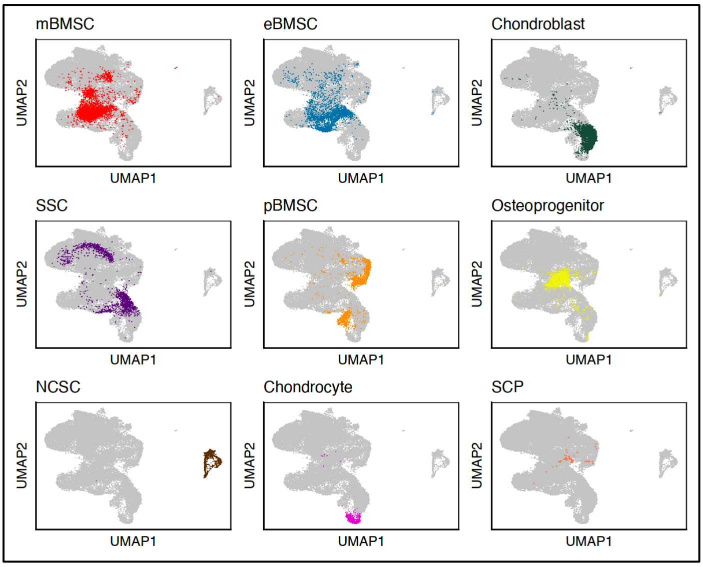
mBMSC
eBMSC
Chondroblast
SSC
pBMSC
Osteoprogenitor
NCSC
Chondrocyte
SCP
UMAP2
UMAP1

Fig. S2

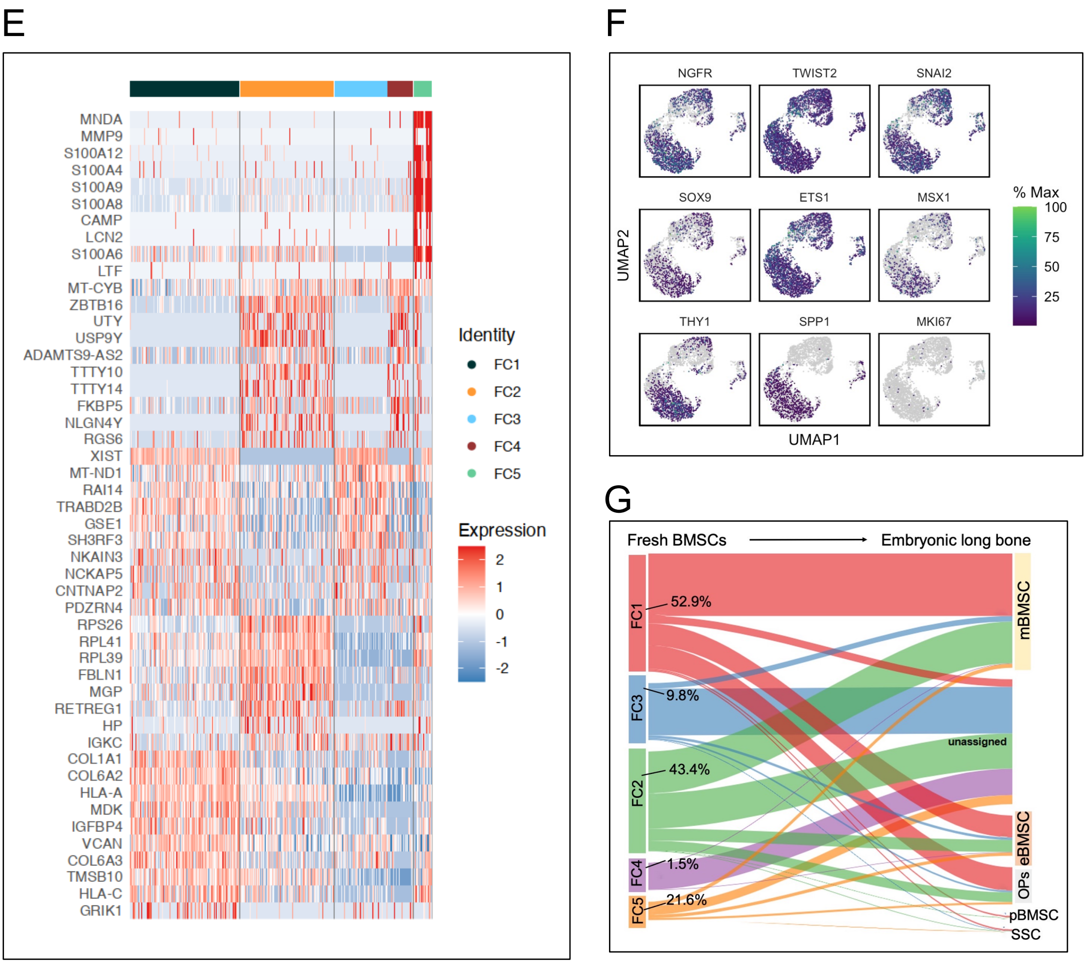
E
MNDA
MMP9
S100A12
S100A4
S100A9
S100A8
CAMP
LCN2
S100A6
LTF
MT-CYB
ZBTB16
UTY
USP9Y
ADAMTS9-AS2
TTTY10
TTTY14
FKBP5
NLGN4Y
RGS6
XIST
MT-ND1
RAI14
TRABD2B
GSE1
SH3RF3
NKAIN3
NCKAP5
CNTNAP2
PDZRN4
RPS26
RPL41
RPL39
FBLN1
MGP
RETREG1
HP
IGKC
COL1A1
COL6A2
HLA-A
MDK
IGFBP4
VCAN
COL6A3
TMSB10
HLA-C
GRIK1
Identity
FC1
FC2
FC3
FC4
FC5
Expression
2
1
0
-1
-2
F
NGFR
TWIST2
SNAI2
SOX9
ETS1
MSX1
THY1
SPP1
MKI67
UMAP2
UMAP1
% Max
100
75
50
25
G
Fresh BMSCs
Embryonic long bone
FC1
52.9%
FC3
9.8%
FC2
43.4%
FC4
1.5%
FC5
21.6%
mBMSC
unassigned
eBMSC
OPs
pBMSC
SSC

Fig. S2

(A) Dot plot showing the mRNA expression levels in 10 clusters identified under unsupervised clustering within human embryonic long bone. (B) Heatmap showing the top 5 differentially expressed genes for each cluster. (C) UMAP visualization of 10 clusters (EC1-10) identified in human embryonic long bone and were assigned to corresponding cell types based on characteristic markers. Subsequent feature plot showing the distribution of mesenchymal markers (PDGFRA, CXCL12), stemness markers (PITX1, FoxP1, Gas1), osteogenesis markers (Runx2, Dlx5, Sox9), chondrogenesis markers (SNORC, Acan, OSR2, CDON), neural crest markers (TWIST1, TWIST2, Pax7, Myc, Nestin, NGFR) and proliferation marker (Mki67) with accordance to each cell type, and with localization to the integrated dataset (D). (E) Heatmap showed the top 10 differentially expressed genes for each of five clusters (FC1-5) identied amongst fresh BMSCs. (F) Feature plot showing marker expression of neural crest markers (NGFR, TWIST2, Snail2, Sox9, ETS1, MSX1) mesenchymal markers (THY1, SPP1), and a proliferation marker (Mki67). (G) Scmap showed that fresh BMSCs had most similarity with mBMSCs, followed by eBMSCs and OPs.

Fig. S3

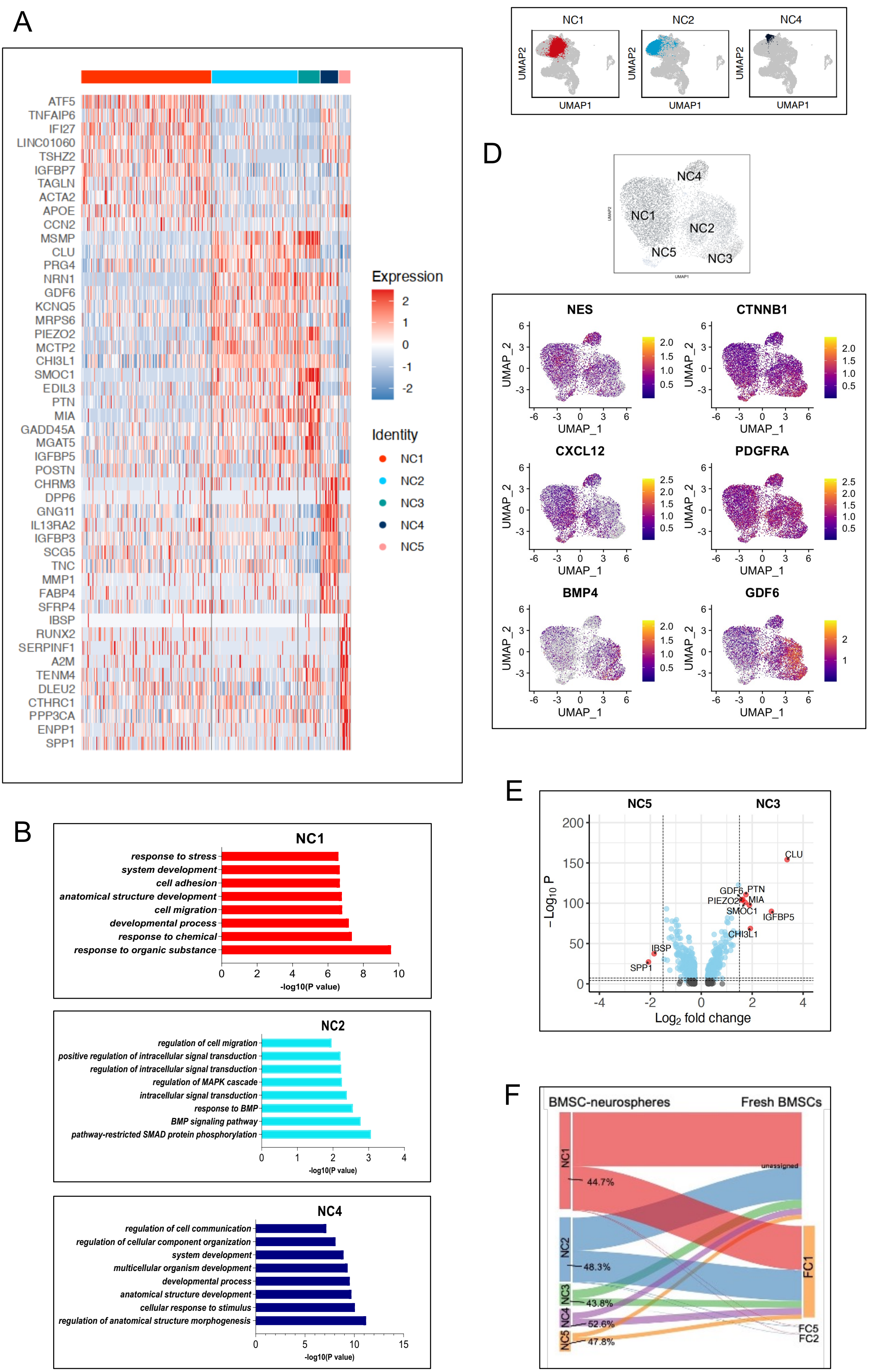
A
ATF5
TNFAIP6
IFI27
LINC01060
TSHZ2
IGFBP7
TAGLN
ACTA2
APOE
CCN2
MSMP
CLU
PRG4
NRN1
GDF6
KCNQ5
MRPS6
PIEZO2
MCTP2
CHI3L1
SMOC1
EDIL3
PTN
MIA
GADD45A
MGAT5
IGFBP5
POSTN
CHRM3
DPP6
GNG11
IL13RA2
IGFBP3
SCG5
TNC
MMP1
FABP4
SFRP4
IBSP
RUNX2
SERPINF1
A2M
TENM4
DLEU2
CTHRC1
PPP3CA
ENPP1
SPP1
Expression
Identity
NC1
NC2
NC3
NC4
NC5
B
NC1
response to stress
system development
cell adhesion
anatomical structure development
cell migration
developmental process
response to chemical
response to organic substance
-log10(P value)
NC2
regulation of cell migration
positive regulation of intracellular signal transduction
regulation of intracellular signal transduction
regulation of MAPK cascade
intracellular signal transduction
response to BMP
BMP signaling pathway
pathway-restricted SMAD protein phosphorylation
-log10(P value)
NC4
regulation of cell communication
regulation of cellular component organization
system development
multicellular organism development
developmental process
anatomical structure development
cellular response to stimulus
regulation of anatomical structure morphogenesis
-log10(P value)
C
NC1
NC2
NC4
UMAP2
UMAP1
D
NC4
NC1
NC2
NC5
NC3
NES
CTNNB1
CXCL12
PDGFRA
BMP4
GDF6
UMAP_2
UMAP_1
E
NC5
NC3
CLU
GDF6
PTN
PIEZO2
MIA
SMOC1
IGFBP5
CHI3L1
IBSP
SPP1
$-\mathrm{Log}_{10}$ P
Log_2 fold change
F
BMSC-neurospheres
Fresh BMSCs
unassigned
NC1
NC2
NC3
NC4
NC5
44.7%
48.3%
43.8%
52.6%
47.8%
FC1
FC5
FC2

Fig. S3

(A) Heatmap showing the top 10 differentially expressed genes for each cluster in the BMSC-neurosphere dataset. (B) Bar charts indicating the results of functional enrichment analysis for NC1, NC2 and NC4 subpopulations. (C) Projections of NC1, NC2 and NC4 upon the integrated dataset. (D) Feature plots of neural and mesenchymal genes expressed upon NC1-5. (E) Volcano plot comparing NC3 and NC5 revealed upregulation of neural-related genes in NC3 (CLU, PTN, GDF6, IGFBP5, and CHI3L1), and osteogenesis-related genes in NC5 (IBSP and SPP1). (F) Scmap showed that BMSC-neurospheres had greatest resemblance to FC1 within fresh BMSCs.
